# Editorial: Women in infectious agents and disease: 2023

**DOI:** 10.3389/fmicb.2024.1436831

**Published:** 2024-06-14

**Authors:** Svetlana Khaiboullina, Ze Chen, Nayeli Alva-Murillo, Alina Maria Holban

**Affiliations:** ^1^Department of Microbiology and Immunology, University of Nevada, Reno, NV, United States; ^2^Hebei Key Laboratory of Animal Physiology, Biochemistry and Molecular Biology, Hebei Collaborative Innovation Center for Eco-Environment, Ministry of Education Key Laboratory of Molecular and Cellular Biology, College of Life Sciences, Hebei Normal University, Shijiazhuang, China; ^3^Division of Natural and Exact Sciences (DCNE), Departament of Biology, University of Guanajuato, Guanajuato, Mexico; ^4^Department of Microbiology and Immunology, Faculty of Biology, University of Bucharest, Bucharest, Romania

**Keywords:** women, science, infection, microbiology, immune defense

A significant gender gap remains in all fields of science, with women publishing and patenting less than men (Mairesse and Pezzoni, 2015; Huang et al., 2020). Additionally, women are less likely to receive an authorship for their contribution to the research (Welle, 2022). Studies have also reported that women's research is cited less compared to that of males (Huang et al., 2020; Madsen et al., 2022). As Huang et al. (2020) stated, it is not only the total number of publications but also the percentage of women's work citations is 30% less compared to that of men. This may greatly affect women's career paths, potentially prompting some to exit the field of science.

To promote the women contribution to science we launched the Research Topic entitled as *Women in infectious agents and disease: 2023*. Seven original articles and two reviews were published on this Research Topic.

The original article by Zhou et al. aimed to conduct the molecular analysis of CAMP-negative *Streptococcus agalactiae* strains. Colonization of the birth canal, a distal reproductive system structure, is the primary mechanism of GBS transmission to a neonate (Miselli et al., 2022), leading to potential severe healthcare problems such as sepsis, meningitis, and pneumonia (Heath and Jardine, 2014). The authors stated that CAMP-negative isolates comprised 7.9% of all GBS isolates. All CAMP-negative strains were missing *cfb* gene coding for CAMP. The authors assert that these findings diverge from earlier observations, where not all CAMP-negative strains lacked the *cfb* gene (Guo et al., 2019; Tickler et al., 2019). This high frequency of gene depletion in CAMP-negative GBS was attributed to using two sets of PCR primers, which could make these results more accurate. There was no correlation between the CAMP negativity and antibiotic resistance.

Kumalo et al. analyzed the abundance of GBS in rectal and vaginal samples from pregnant women in Ethiopia. The prevalence of GBS in collected samples was 24%, falling within the globe range (Arain et al., 2015; Kwatra et al., 2016; Nishihara et al., 2017). The authors indicated no association between GBS colonization and socio-demographic data. However, there was a positive correlation between a college or above level of education and GBS colonization. An important observation was finding the high number of isolates resistant to tetracycline, ciprofloxacin, and clindamycin. The authors emphasize the importance of screening pregnant women for GBS and performing antibiotic susceptibility tests prior to selecting treatment.

Several studies have shown that a disturbed vaginal microbiome could be a risk factor for cervical cancer. To address this hypothesis, Frąszczak et al. sought to analyze the distribution of *Lactobacillus* spp. in women with abnormal Pap smear results in controls among Polish women. They found that *Lactobacillus* spp. did not differ between vaginal smear samples. However, *L. acidophilus* and *L. fermentum* were more frequent in samples collected from women in rural areas compared to urban areas. Interestingly, there were no differences in *Lactobacillus* spp. among HPV-positive and negative patients and in patients with bacterial infection. The analysis revealed a positive correlation between *L. delbrueckii* and *L. gasseri*, as well as a negative correlation between *L. fermentum* and *L. plantarum* in patients with abnormal Pap smear results.

Genital schistosomiasis is a vector-borne neglected tropical disease often diagnosed in tropical countries (Colley et al., 2014). In a study by Rausche et al., the awareness of schistosomiasis among the risk population was analyzed in Madagascar. There was higher awareness of schistosomiasis among HCWs (53.8%), while it was lower within the general population of women (11.3%). Also, the lowest awareness was among young (18–25 years old) and older (45+ years old) compared to other age groups. Family members were the primary source of knowledge among women. The authors emphasize the necessity of raising awareness about schistosomiasis among women to control this neglected tropical disease.

Tuberculosis (TB) remains a serious healthcare concern in many countries (WHO, 2013). Pathogenesis of TB includes macrophages serving as a primary site of microbial persistence (Cumming et al., 2018) which is achieved by utilizing nutrient resources such as carbohydrates, amino acids, and lipids as well as modulation of metabolic pathways favoring *Mycobacterium tuberculosis* (Mtb) propagation within the cell (Beste et al., 2013; Cumming et al., 2018; Borah et al., 2019). Slater et al. analyzed the intracellular carbon metabolic fluxes in Mtb-infected macrophages. An increased glycolytic flux toward pyruvate synthesis and reduced pentose phosphate pathway were found in infected macrophages compared to controls. The TCA pathway was inhibited in Mtb-infected THP-1 cells. Infected cells exhibited decreased levels of serine, glycine, and cysteine, while experiencing increased synthesis fluxes for aspartate, glutamine, and glutamate in macrophages. The authors state that identified metabolic changes in Mtb-infected macrophages could be targeted for developing novel therapeutics for TB.

The study by Wu et al. was aimed to analyze the role of SR2 in the pathogenesis of *Toxoplasma gondii*. Using the CRISPR-Cas9 gene editing approach, the authors identified and functionally characterized SR2, revealing its localization in the nucleus and expression only in the tachyzoite and bradyzoite stages. Additionally, the authors demonstrated that the deletion of SR2 in the type I RH strain and type II Pru strain of *T. gondii* had a limited effect on growth and bradyzoite differentiation. The disruption of this gene resulted in attenuation of the microbial virulence. The authors state that SR2 plays a role in the pathogenicity of *T. gondii* and could be a promising target for novel therapeutics against toxoplasmosis.

Chronic venous ulcer of the lower limb is a complication developed in patients with advanced venous disease (Stanek et al., 2023). *Staphylococcus aureus* is the most frequently identified microflora in patients with chronic venous ulcers (Gajda et al., 2021). However, our knowledge of virulence and resistance of strains from venous ulcer patients is limited. In the present study, Mihai et al. aimed to characterize the phenotypic virulence profiles of *S. aureus* isolated from chronic skin wounds and complete the correlation analysis with clinical presentation. The most common bacterial species was *S. aureus* capable developing a biofilm and producing toxins. The authors suggest that early analysis of bacteria linked to chronic ulcers could aid in tailoring personalized treatments for the disease.

Two review papers were published in this Research Topic.

In the first review, Patel and Rawat summarize the current knowledge on *S. aureus* MRSA pathogenesis. The authors state that biofilm formation is essential in the pathogenesis of MRSA. Biofilm formation is maintained by the expression of polysaccharide intercellular adhesin, extracellular DNA, teichoic acids, and capsule and virulence factors. These virulence factors are transcriptionally regulated by accessory gene regulator (*agr*) and *S. aureus* exoprotein expression (*sae*) locus. *Agr* regulates quorum sensing, increases virulence factor secretion, and contributes to MRSA pathogenesis *in vivo* (Bunce et al., 1992). These virulence factors could also contribute to the evasion of the immune response by this microbe. This modulation of virulence factors expression is a genetic regulatory “see-saw” of *S. aureus* pathogenesis.

In the second review, Wojciechowska et al. focused on the importance of fungi in the microbiome of neonates in the intensive care unit. The foremost important source of neonatal microbiome is that of the mother: endometrial, vaginal, gastrointestinal, and oral (Mueller et al., 2015; Yao et al., 2021). Maternal microbiome could be affected by genetics, diet, medications, infections, and stress (Cahana and Iraqi, 2020; Patangia et al., 2022; Galley et al., 2023). Additionally, the microbiome of neonates could be affected by gestation age at birth and breast milk biota (Boudry et al., 2021; Arboleya et al., 2022). The authors state that most of the research focuses on bacterial components of neonatal microbiome. However, changes in fungi species are often neglected. Studies on fungi in neonatal microbiome are urgently needed as 13% of gut microbes are fungi (Schei et al., 2017). Fungal infections remain a leading cause of morbidity and mortality in preterm neonates (Hsieh et al., 2012). The authors address the current gaps in our understanding of the role of fungi in disturbed neonatal microbiome. The importance of personalized medicine is acknowledged in this review as one of the approaches for the treatment of fungal infections in neonates.

We thank the authors and reviewers for their valuable contributions to the research and insights.

## Author contributions

SK: Conceptualization, Writing – original draft. ZC: Formal analysis, Writing – review & editing. NA-M: Writing – review & editing, Software. AH: Validation, Writing – review & editing.

## References

[B1] Arain F. R. Al-Bezrah N. A. Al-Aali K. Y. (2015). Prevalence of maternal genital tract colonization by group B *Streptococcus* from Western Province, Taif, Saudi Arabia. J. Clin. Gynecol. Obstet. 4, 258–264. 10.14740/jcgo341w

[B2] Arboleya S. Rios-Covian D. Maillard F. Langella P. Gueimonde M. Martín R.. (2022). Preterm delivery: microbial dysbiosis, gut inflammation and hyperpermeability. Front. Microbiol. 12:806338. 10.3389/fmicb.2021.806338 35185831 PMC8854986

[B3] Beste D. J. Nöh K. Niedenführ S. Mendum T. A. Hawkins N. D. Ward J. L.. (2013). 13C-flux spectral analysis of host-pathogen metabolism reveals a mixed diet for intracellular *Mycobacterium tuberculosis*. Chem. Biol. 20, 1012–1021. 10.1016/j.chembiol.2013.06.012 23911587 PMC3752972

[B4] Borah K. Girardi K. C. V. Mendum T. A. Lery L. M. S. Beste D. J. Lara F. A.. (2019). Intracellular *Mycobacterium leprae* utilizes host glucose as a carbon source in Schwann cells. mBio 10:e02351-19. 10.1128/mBio.02351-19 31848273 PMC6918074

[B5] Boudry G. Charton E. Le Huerou-Luron I. Ferret-Bernard S. Le Gall S. Even S.. (2021). The relationship between breast milk components and the infant gut microbiota. Front. Nutr. 8:629740. 10.3389/fnut.2021.629740 33829032 PMC8019723

[B6] Bunce C. Wheeler L. Reed G. Musser J. Barg N. (1992). Murine model of cutaneous infection with gram-positive cocci. Infect. Immun. 60, 2636–2640. 10.1128/iai.60.7.2636-2640.1992 1612733 PMC257214

[B7] Cahana I. Iraqi F. A. (2020). Impact of host genetics on gut microbiome: take-home lessons from human and mouse studies. Animal Model Exp. Med. 3, 229–236. 10.1002/ame2.12134 33024944 PMC7529332

[B8] Colley D. G. Bustinduy A. L. Secor W. E. King C. H. (2014). Human schistosomiasis. Lancet 383, 2253–2264. 10.1016/S0140-6736(13)61949-2 24698483 PMC4672382

[B9] Cumming B. M. Addicott K. W. Adamson J. H. Steyn A. J. (2018). *Mycobacterium tuberculosis* induces decelerated bioenergetic metabolism in human macrophages. Elife 7:e39169. 10.7554/eLife.39169.018 30444490 PMC6286123

[B10] Gajda M. Załugowicz E. Pomorska-Wesołowska M. Bochenek T. Gryglewska B. Romaniszyn D.. (2021). Virulence and drug-resistance of *Staphylococcus aureus* strains isolated from venous ulcers in polish patients. Int. J. Environ. Res. Public Health 18:4662. 10.3390/ijerph18094662 33925700 PMC8124697

[B11] Galley J. D. Mashburn-Warren L. Blalock L. C. Lauber C. L. Carroll J. E. Ross K. M.. (2023). Maternal anxiety, depression and stress affects offspring gut microbiome diversity and bifidobacterial abundances. Brain Behav. Immun. 107, 253–264. 10.1016/j.bbi.2022.10.005 36240906

[B12] Guo D. Xi Wang Y. Wang S. Z. (2019). Is a positive Christie-Atkinson-Munch-Peterson (CAMP) test sensitive enough for the identification of *Streptococcus agalactiae*? BMC Infect. Dis. 19, 1–5. 10.1186/s12879-018-3561-3 30606123 PMC6318942

[B13] Heath P. T. Jardine L. A. (2014). Neonatal infections: group B *Streptococcus*. BMJ Clin. Evid. 2014:0323. PMC3938141 24580886

[B14] Hsieh E. Smith P. B. Jacqz-Aigrain E. Kaguelidou F. Cohen-Wolkowiez M. Manzoni P.. (2012). Neonatal fungal infections: when to treat? Early Hum. Dev. 88, S6–S10. 10.1016/S0378-3782(12)70004-X 22633516 PMC3512570

[B15] Huang J. Gates A. J. Sinatra R. Barabási A.-L. (2020). Historical comparison of gender inequality in scientific careers across countries and disciplines. Proc. Nat. Acad. Sci. 117, 4609–4616. 10.1073/pnas.1914221117 32071248 PMC7060730

[B16] Kwatra G. Cunnington M. C. Merrall E. Adrian P. V. Ip M. Klugman K. P.. (2016). Prevalence of maternal colonisation with group B *Streptococcus*: a systematic review and meta-analysis. Lancet Infect. Dis. 16, 1076–1084. 10.1016/S1473-3099(16)30055-X 27236858

[B17] Madsen E. B. Nielsen M. W. Bjørnholm J. Jagsi R. Andersen J. P. (2022). Author-level data confirm the widening gender gap in publishing rates during COVID-19. Elife 11:e76559. 10.7554/eLife.76559.sa2 35293860 PMC8942470

[B18] Mairesse J. Pezzoni M. (2015). Does gender affect scientific productivity? A critical review of the empirical evidence and a panel data econometric analysis for French physicists. Rev. Écon. 66, 65–113. 10.3917/reco.661.006518052372

[B19] Miselli F. Frabboni I. Di Martino M. Zinani I. Buttera M. Insalaco A.. (2022). Transmission of Group B *Streptococcus* in late-onset neonatal disease: a narrative review of current evidence. Ther. Adv. Infect. Dis. 9, 20499361–221142732. 10.1177/20499361221142732 36569815 PMC9780763

[B20] Mueller N. T. Bakacs E. Combellick J. Grigoryan Z. Dominguez-Bello M. G. (2015). The infant microbiome development: mom matters. Trends Mol. Med. 21, 109–117. 10.1016/j.molmed.2014.12.002 25578246 PMC4464665

[B21] Nishihara Y. Dangor Z. French N. Madhi S. Heyderman R. (2017). Challenges in reducing group B *Streptococcus* disease in African settings. Arch. Dis. Child. 102, 72–77. 10.1136/archdischild-2016-311419 27831912 PMC5256401

[B22] Patangia D. V. Anthony Ryan C. Dempsey E. Paul Ross R. Stanton C. (2022). Impact of antibiotics on the human microbiome and consequences for host health. Microbiologyopen 11:e1260. 10.1002/mbo3.1260 35212478 PMC8756738

[B23] Schei K. Avershina E. Øien T. Rudi K. Follestad T. Salamati S.. (2017). Early gut mycobiota and mother-offspring transfer. Microbiome 5, 1–12. 10.1186/s40168-017-0319-x 28837002 PMC5571498

[B24] Stanek A. Mosti G. Nematillaevich T. S. Valesky E. M. Planinšek Ručigaj T. Boucelma M.. (2023). No more venous ulcers—what more can we do? J. Clin. Med. 12:6153. 10.3390/jcm12196153 37834797 PMC10573394

[B26] Tickler I. A. Tenover F. C. Dewell S. Le V. M. Blackman R. N. Goering R. V.. (2019). *Streptococcus agalactiae* strains with chromosomal deletions evade detection with molecular methods. J. Clin. Microbiol. 57:e02040-18. 10.1128/JCM.02040-18 30760532 PMC6440789

[B25] Welle E. (2022). Women less likely than men to get authorship on scientific publications, analysis finds. Avilable online at: https://www.statnews.com/2022/06/22/women-less-likely-than-men-to-get-authorship-on-scientific-publications-analysis-finds/#:~:text=Specifically%2C%20women%20are%2013%25%20less,receive%20credit%20on%20a%20patent (accessed June 6, 2024).

[B27] WHO (2013). Global Tuberculosis Report 2013. Geneva: World Health Organization.

[B28] Yao Y. Cai X. Ye Y. Wang F. Chen F. Zheng C.. (2021). The role of microbiota in infant health: from early life to adulthood. Front. Immunol. 12:708472. 10.3389/fimmu.2021.708472 34691021 PMC8529064

